# Identifying circRNA–miRNA–mRNA Regulatory Networks in Chemotherapy-Induced Peripheral Neuropathy

**DOI:** 10.3390/cimb45080430

**Published:** 2023-08-16

**Authors:** Fei Cao, Xintong Wang, Qingqing Ye, Fang Yan, Weicheng Lu, Jingdun Xie, Bingtian Bi, Xudong Wang

**Affiliations:** 1 Department of Anesthesiology, State Key Laboratory of Oncology in South China, Sun Yat-sen University Cancer Center, Guangzhou 510060, China; caof2@sysucc.org.cn (F.C.); wangxt37@mail2.sysu.edu.cn (X.W.); yeqq@sysucc.org.cn (Q.Y.); yanfang@sysucc.org.cn (F.Y.); luwc@sysucc.org.cn (W.L.); xiejd@sysucc.org.cn (J.X.); 2 Department of Clinical Trial Center, State Key Laboratory of Oncology in South China, Sun Yat-sen University Cancer Center, Guangzhou 510060, China

**Keywords:** chemotherapy-induced peripheral neuropathy, circular RNA, ceRNA, immune infiltration, regulatory network

## Abstract

Chemotherapy-induced peripheral neuropathy (CIPN) is a frequent and severe side effect of first-line chemotherapeutic agents. The association between circular RNAs (circRNAs) and CIPN remains unclear. In this study, CIPN models were constructed with Taxol, while 134 differentially expressed circRNAs, 353 differentially expressed long non-coding RNAs, and 86 differentially expressed messenger RNAs (mRNAs) were identified utilizing RNA sequencing. CircRNA-targeted microRNAs (miRNAs) were predicted using miRanda, and miRNA-targeted mRNAs were predicted using TargetScan and miRDB. The intersection of sequencing and mRNA prediction results was selected to establish the circRNA–miRNA–mRNA networks, which include 15 circRNAs, 18 miRNAs, and 11 mRNAs. Functional enrichment pathway analyses and immune infiltration analyses revealed that differentially expressed mRNAs were enriched in the immune system, especially in T cells, monocytes, and macrophages. *Cdh1*, *Satb2*, *Fas*, *P2ry2*, and *Zfhx2* were further identified as hub genes and validated by RT-qPCR, correlating with macrophages, plasmacytoid dendritic cells, and central memory CD4 T cells in CIPN. Additionally, we predicted the associated diseases, 36 potential transcription factors (TFs), and 30 putative drugs for hub genes using the DisGeNET, TRRUST, and DGIdb databases, respectively. Our results indicated the crucial role of circRNAs, and the immune microenvironment played in CIPN, providing novel insights for further research.

## 1. Introduction

Chemotherapy-induced peripheral neuropathy (CIPN) is a frequent and severe long-term adverse effect of several first-line chemotherapeutic agents, including paclitaxel. The neurotoxicity of paclitaxel impairs the dorsal horn of spinal cords, which can lead to paresthesia and mechanical allodynia [1]. Up to 40% of cancer survivors may have lifelong symptoms and incapacity as a result of CIPN [2], resulting in a significant economic burden on patients and the healthcare system [3]. However, no proven strategy is currently available to prevent or limit the occurrence of CIPN [4], and further in-depth studies are required to elucidate the underlying mechanisms.

CircRNAs are characterized by single-stranded, covalently closed RNA molecules without 5′ or 3′ ends [5]. They have a different structure from linear RNAs, which gives them a longer half-life and greater resistance to RNase R, making them satisfactory options for therapeutic targets and diagnostic biomarkers [6]. According to previous studies, circRNAs could interface precisely with DNA and RNA, regulate mRNA’s stability and translation as competitive endogenous RNAs (ceRNAs), interact on signaling pathways, and even serve as templates in protein translation [7]. CircRNAs have recently been reported to mediate inflammation factors such as IL-1, IL-10, and TNF-α, which are significant in the progression of neuropathic pain [8]. *CircSMEK1* facilitates neuropathic pain inflammation by modulating the *miR-216a-5p*/*TXNIP* axis [9]. The *circKat6b*/*mi26a*/*Kcnk1* pathway in the dorsal horn regulates the development and maintenance of neuropathic pain [10]. CircRNA–miRNA–mRNA regulatory network has also been reported to engage in paclitaxel-induced CIPN [11]. However, the potential roles of circRNAs in CIPN are not well understood.

**Figure 1:**
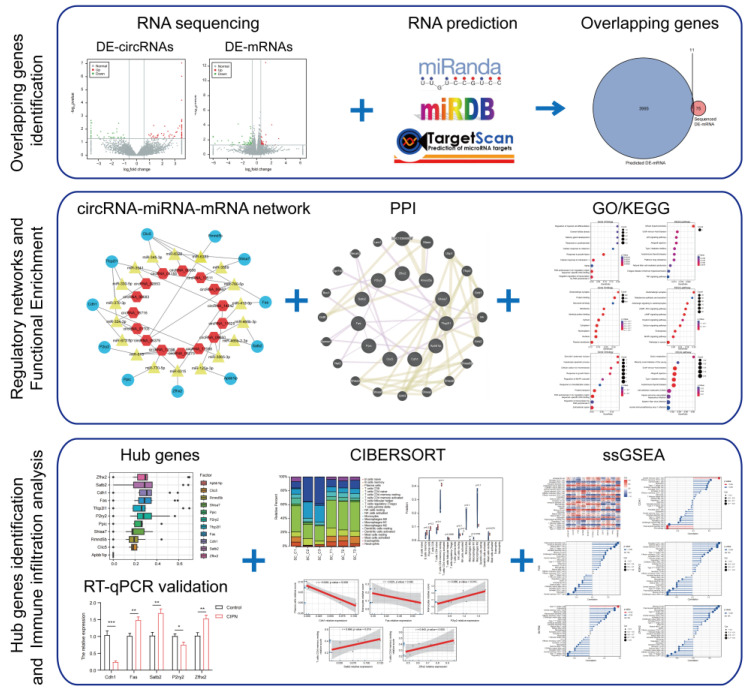

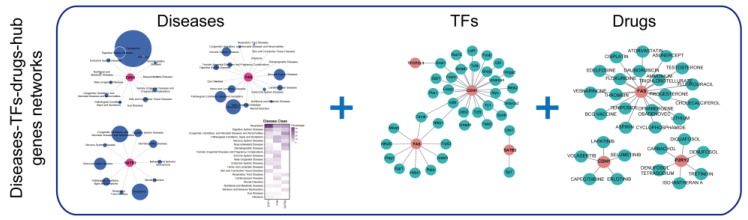
Flowchart of this study. The overlapping genes between DE-mRNAs and predicted mRNAs regulated by DE-circRNAs-targeted miRNAs were obtained first. CeRNA networks, PPI networks, and functional enrichment analyses were then performed. Next, hub genes were selected and validated by RT-qPCR. The immune infiltration analysis, immune cell correlation, and prediction of associated diseases, potential TFs, and effective drugs were also conducted. * *p* < 0.05; ** *p* < 0.01; *** *p* < 0.001.

In this work, we explored the differentially expressed genes (DEGs) in taxol-treated rats and control rats using sequencing of non-coding RNAs combined with our previous sequencing data (Figure 1). Next, we focused on the regulatory mechanisms of circRNAs. Subsequently, we constructed circRNA–miRNA–mRNA networks, performed functional enrichment pathway analyses, and identified and validated hub genes to elucidate the pathogenesis and underlying molecular mechanisms of CIPN. The results of the immune infiltration analysis also provided important information on the role of immunity in CIPN. Potential TFs and effective drugs were predicted to better understand the mechanisms and treatment of CIPN.

## 2. Materials and Methods

### 2.1. Animals

Experiments were carried out in adult male Sprague–Dawley rats weighing between 180 and 250 g. The rats were obtained from the Guangdong Medical Laboratory Center, China, and were kept in cages with a 12/12 h light/dark cycle and unlimited access to food and water. The Animal Care and Use Committee of the Sun Yat-sen University Cancer Center gave all experimental procedures its approval (L102022020004A). This study followed the National Institutes of Health guidelines for ethical treatment and the care of animals, and strictly conformed to the guidelines for the caring and utilization of laboratory animals.

### 2.2. Paclitaxel-Induced Peripheral Neuropathy Model

Rats were divided into two groups at random: vehicle (three rats) and paclitaxel treatment (three rats). Paclitaxel (2 mg/kg; Taxol, C47H51NO14, Bristol Myers Squibb, Princeton, NJ, USA) was injected intraperitoneally on four alternate days (days 1, 3, 5, and 7), bringing about a cumulative dose of 8 mg/kg per rat, to induce peripheral neuropathy as previously described [12]. The intraperitoneal injection of 0.9% saline was given to the control groups in the same quantity.

### 2.3. Collection of Tissues, Extraction of RNA, Creation of Library, and Sequencing

On day 10, sodium pentobarbital (100 mg/kg, i.p.) with 0.9% saline was transcardially perfused to anesthetize the rats at 4 °C. Immediately after perfusion; we obtained the spinal cord dorsal horns, which were involved in the transmission and maintenance of pain information in CIPN. Sequencing and library creation were performed by OE Biotech Co., Ltd. Using the mirVana miRNA Isolation Kit (Ambion), RNAs were isolated from randomly chosen control tissues (*n* = 3) and paclitaxel group tissues (*n* = 3). The DNAs remaining in the tissues were digested using DNase I, whereas the eluate obtained after spinning was the total RNA. The RNA integrity was assessed with the Agilent 2100 Bioanalyzer (Agilent Technologies, Santa Clara, CA, USA). Samples with an RNA Integrity Number (RIN) ≥ 7 were chosen for the following analysis. The TruSeq Stranded Total RNA with Ribo-Zero Gold kit and Illumina HiSeq 2500 platform was utilized to generate 150 bp/125 bp paired-end reads and construct the libraries.

### 2.4. Differentially Expressed RNAs Identification

The DESeq2 R package was utilized to identify statistically significant differentially expressed circRNAs (DE-circRNAs), lncRNAs, and mRNAs (DE-mRNAs) [13]. All the expression values were changed into log2 values. The screening criteria for DE-circRNAs and DE-mRNAs were *p*-value < 0.05 and |log2(fold-change)| > 0.58. Heatmaps and volcano plots were generated utilizing the R heatmap and ggplot2 packages to visualize the observed differences.

### 2.5. Establishment of the circRNA-miRNA-mRNA Networks and Protein–Protein Interaction (PPI) Network

CircRNA-miRNA interactions were predicted using the miRanda database (http://www.microrna.org/, accessed on 25 April 2023), while the interactions between mRNAs and miRNAs were analyzed by intersecting the results from the TargetScan (http://www.targetscan.org/, accessed on 10 May 2023) and miRDB databases (http://www.mirdb.org/, accessed on 10 May 2023). The resulting circRNA-miRNA-mRNA network was visualized using Cytoscape 3.8.2. Additionally, the GeneMANIA database (http://genemania.org/, accessed on 15 June 2023) was utilized to build a protein–protein interaction (PPI) network for DE-mRNAs. To evaluate the correlation between DE-circRNAs and DE-mRNAs, Spearman’s correlation analysis was performed.

### 2.6. Functional Enrichment Pathway Analysis

To portray the function of DE-circRNAs and DE-mRNAs, we conducted Gene Ontology (GO) and the Kyoto Encyclopedia of Genes and Genomes (KEGG) pathway based on the predicted mRNAs, DE-mRNAs, and overlapping mRNAs. The enrichment analysis was performed utilizing the clusterProfiler R package [14]. The 10 highest-ranked terms from GO and KEGG analyses with a *p*-value < 0.05 were selected.

### 2.7. Tissue-Most Expressed Gene Analysis and Hub Genes Identification

We identified the systems/tissues with the highest expression levels of the overlapping mRNAs in CIPN by exploring their distribution using the online resource BioGPS (http://biogps.org/, accessed on 24 June 2023). We then utilized the GOSemSim R package to perform a Friends analysis, which computed the similitudes among genes and ranked them in light of their GO terms and interactions with each other [15]. A higher correlation index and comparability suggested more prominent importance. Five genes with the highest results were selected as hub genes.

### 2.8. Immune Infiltration Analysis

The CIBERSORT algorithm and the single-sample gene set enrichment analysis (ssGSEA) were used to decode the immune infiltration microenvironment in the CIPN rat model. CIBERSORT provides an estimate of immune cell abundance in a mixed cell population. At the same time, ssGSEA is a position-based technique that scores the absolute enrichment of a particular gene set in each sample. The correlation between immune cells and DE-mRNAs was assessed using Spearman’s correlation analysis.

### 2.9. Diseases, TFs, and Drugs Prediction of Hub Genes

We utilized the disgenet2r R package to retrieve gene-disease associations of hub genes from DisGeNET (https://www.disgenet.org/, accessed on 3 July 2023) and grouped the diseases using the MeSH Disease Class [16]. For predicting potential transcription regulators of the hub genes, we employed TRRUST (https://www.grnpedi.org/trrust/, accessed on 13 July 2023). We used the drug prediction database DGIdb (https://dgidb.org/, accessed on 13 July 2023) to anticipate drugs for the hub genes. Subsequently, we downloaded the results to obtain the disease-hub genes, TF-hub genes, and drug-hub genes interaction networks. Finally, we utilized Cytoscape software 3.8.2 to visualize the graph.

### 2.10. Validation of Hub Genes Expression

The spinal cord dorsal horns were collected from randomly chosen control tissues (*n* = 6) and paclitaxel group tissues (*n* = 6). The total RNA was reverse transcribed into complementary DNA (cDNA) utilizing the PrimeScript RT reagent kit (Takara, Tokyo, Japan). SYBR Premix Ex Taq (Takara, Tokyo, Japan) and primers targeting five hub genes (Table S1) were added to cDNA mixtures for RT-qPCR. Gapdh was chosen as a control housekeeping gene. The relative expressions of hub mRNAs were analyzed with the 2^−ΔΔ^cycle threshold (CT) method.

### 2.11. Statistical Analysis

All experimental data were analyzed and expressed as mean ± standard error of the mean using GraphPad Prism 8.0. Comparisons of mRNA expressions by RT-qPCR among two groups were evaluated using the unpaired Student’s *t*-test. All statistical significance was set at *p*-value < 0.05 in this study.

## 3. Results

### 3.1. Profile of Differentially Expressed Genes in CIPN

**Figure 2:**
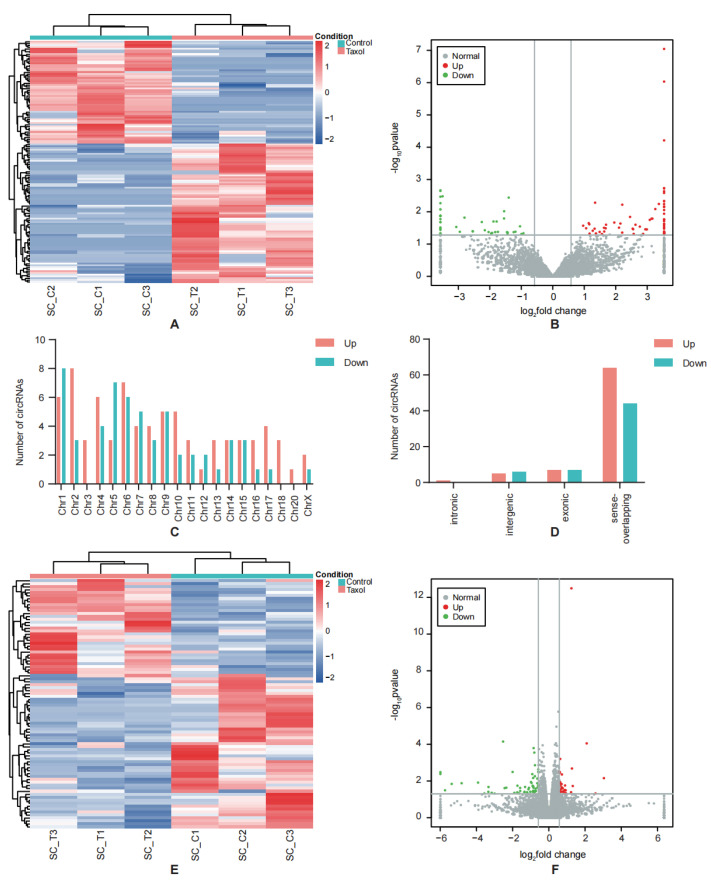
DE-circRNAs identification. (**A**) The heatmap of DE-circRNAs indicates the differentially expressed circRNAs between CIPN and control samples. (**B**) Volcano plot of all DE-circRNAs. (**C**) Chromosomal distributions of DE-circRNAs in CIPN and control samples. (**D**) Statistics of DE-circRNAs types. (**E**) The heatmap of DE-mRNAs indicates the differentially expressed mRNAs between CIPN and control samples. (**F**) Volcano plot of all DE-mRNAs. Green dots indicate the downregulated ones, and red dots indicate the upregulated ones.

Three sets of spinal dorsal horns from rats with CIPN and normal rats were analyzed using RNA-seq to identify the expression profiles of RNAs in CIPN tissues. Combined with the previously published data about mRNA profiles in CIPN [17], We then conducted a differential expression analysis, setting the significance threshold as |log2(foldchange)| > 0.58 and a *p*-value < 0.05. Using hierarchical clustering analysis, the 134 DE-circRNAs’ expression patterns were identified (Figure 2A). Among these circRNAs, 77 were upregulated, and 57 were downregulated in CIPN tissues compared to normal tissues (Figure 2B). The distribution of DE-circRNAs in the chromosomes and their types are shown in Figure 2C,D. The top 20 upregulated and 20 downregulated circRNAs are shown in Table 1 and Table 2, indicating that they are more likely to play a greater role in the regulation of CIPN. Meanwhile, 353 lncRNAs were identified as differentially expressed (Figure S1), and 86 DE-mRNAs were identified (Figure 2E,F). Since many studies have explored the contribution of lncRNAs to neuropathic pain [18], we focused on the possible roles of circRNAs.

### 3.2. Construction of circRNA-miRNA-mRNA Networks and Protein–Protein Interaction Networks

**Figure 3:**
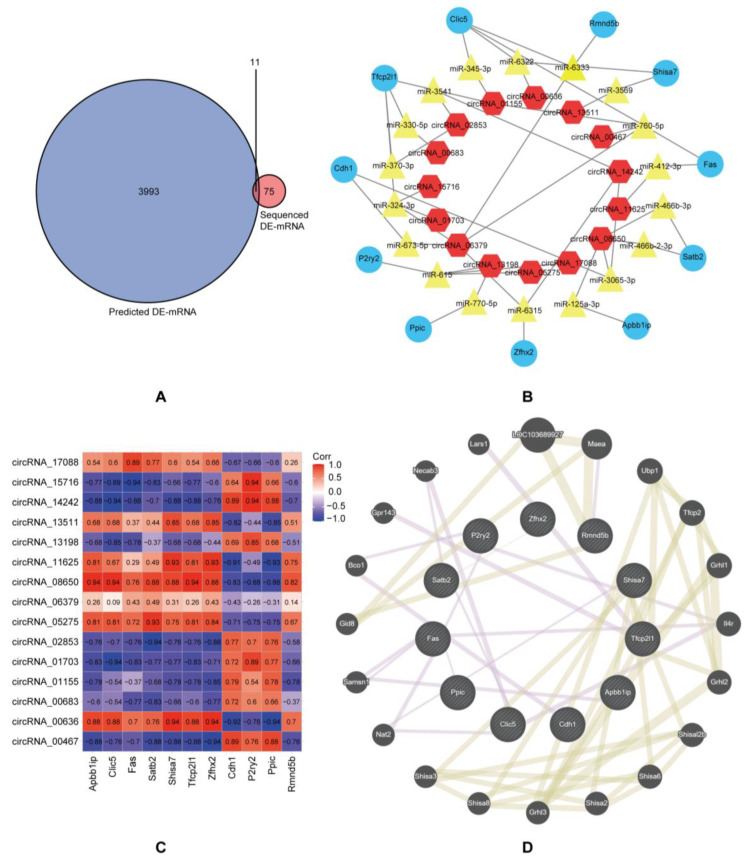
The circRNA-miRNA-mRNA regulatory network. (**A**) Venn diagram, sequenced DE-mRNAs in red, predicted DE-mRNAs in blue. (**B**) Representative circRNA-miRNA-mRNA network constructed by Cytoscape software 3.8.2. Blue dots indicate mRNAs, yellow dots indicate miRNAs, and red dots indicate circRNAs. (**C**) Correlations between DE-circRNAs and DE-mRNAs in CIPN. (**D**) Overlapping mRNAs and their co-expression genes were analyzed using the GeneMANIA database. Each node represents a gene. The purple lines represent the type of network interaction that is co-expression, and the yellow lines represent shared protein domains.

**Figure 4:**
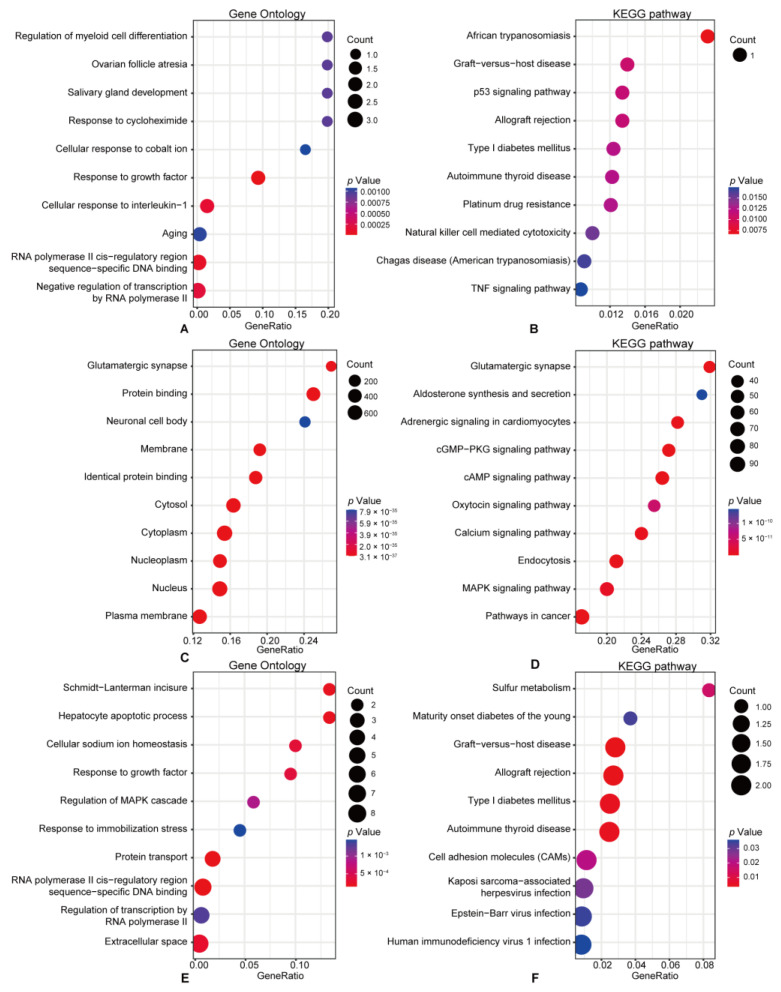
GO and KEGG analyses of the DE-mRNAs. (**A**,**B**) GO analysis and KEGG pathways analysis of the overlapping mRNAs. (**C**,**D**) GO analysis and KEGG pathways analysis of the predicted mRNAs. (**E**,**F**) GO analysis and KEGG pathways analysis of the DE-mRNAs between the CIPN and control tissues.

Because circRNAs mainly perform their biological functions as competing endogenous RNAs (ceRNAs) to regulate gene expression by sponging miRNAs [8], we predicted miRNAs that may bind to DE-circRNAs using miRanda (Table S2). We identified mRNAs that the above-mentioned miRNAs might target using TargetScan and miRDB. To further explore the potential CIPN-related mRNAs, an intersection was conducted between the predicted and DE-mRNAs (Figure 3A). We obtained 11 overlapping mRNAs that may have a stronger correlation with CIPN (Table 3), providing direction for further identification of key genes and the construction of circRNA-miRNA-mRNA regulatory networks in CIPN. Based on the above results, circRNA-miRNA-mRNA regulatory networks containing 15 circRNAs, 18 miRNAs, and 11 overlapping mRNAs were constructed (Figure 3B). We also performed Spearman correlation analyses to help demonstrate a significant correlation between circRNAs and mRNAs in the circRNA-miRNA-mRNA regulatory network (Figure 3C). The GeneMANIA database was utilized to predict the genes connected with the 11 overlapping mRNAs and construct PPI networks. The interaction within the overlapping genes is also shown in Figure 4D. The results showed that 20 genes were linked to interactions with the overlapping mRNAs, and the top 10 predicted genes were *LOC103689927*, *Maea*, *Ubp1*, *Tfcp2*, *Grhl1*, *Il4r*, *Grhl2*, *Shisal2b*, *Shisa6*, and *Shisa2*.

### 3.3. Functional Enrichment Pathway Analysis

To maximize the exploration of regulatory mechanisms and identify the key biological process, we conducted GO and KEGG enrichment analyses on the overlapping mRNAs, predicted mRNAs, and DE-mRNAs using the clusterProfiler R package. Results with a *p*-value < 0.05 were indicated as significant. The 11 overlapping mRNAs were principally enriched in the regulation of myeloid cell differentiation, response to cycloheximide, cellular response to interleukin-1, p53 signaling pathway, and natural killer cell-mediated cytotoxicity (Figure 4A,B). Meanwhile, the mRNAs predicted to be regulated by the DE-circRNAs were mainly advanced in the glutamatergic synapse, neuronal cell body, and MAPK signaling pathways (Figure 4C,D). Further functional enrichment analyses indicated that the DE-mRNAs between CIPN and control tissues were principally associated with Schmidt-Lanterman incisure, hepatocyte apoptotic process, sulfur metabolism, and diabetes (Figure 4E,F). The difference between overlapping mRNAs and DE-mRNAs in functional analysis enrichment confirmed that we had successfully excluded the majority of DE-mRNAs that were unrelated to CIPN, which also helped us narrow our attention on potential critical processes.

### 3.4. Hub Genes Identification and Validation

**Figure 5:**
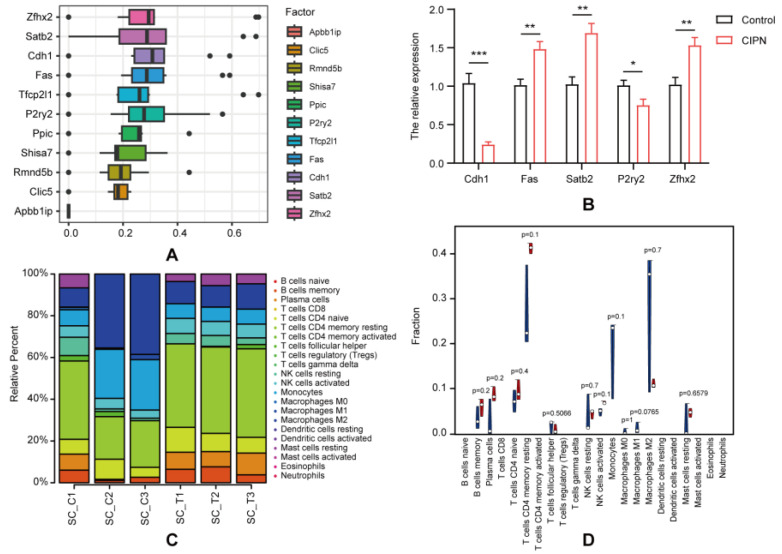

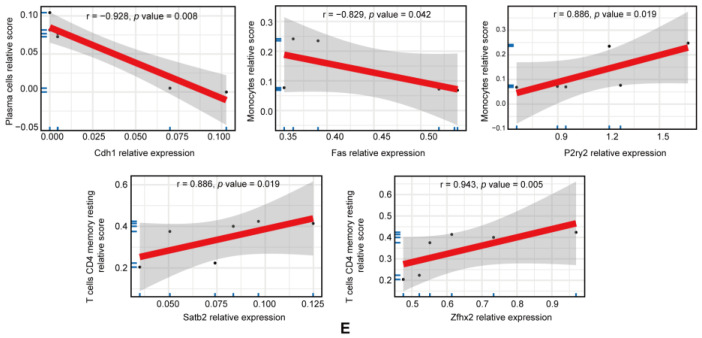
Hub genes identification and immune infiltration analysis. (**A**) Distributions of functional similarities of overlapping mRNAs were summarized as boxplots using GOSemSim. (**B**) The expression level of hub genes Cdh1, Fas, Satb2, P2ry2, and Zfhx2 in the spinal dorsal horn of CINP rats compared with the control validating by RT-qPCR. * *p* < 0.05; ** *p* < 0.01; *** *p* < 0.001 (**C**) The fraction of 22 subsets of immune cells in CIPN and control samples. (**D**) The violin graph shows the difference in immune infiltration between CIPN and control samples. The control samples are shown in blue, and the CIPN samples are shown in red. (**E**) Correlations between hub genes and immune cell infiltration.

BioGPS is an online tool that allows researchers to access distributed gene annotation resources [19]. To assess the likelihood and underlying mechanisms by which genes play a role in CIPN, we used BioGPS to identify the distribution of the 11 overlapping genes. Most of the overlapping genes (36.4%, 4/11) were present in the hematologic/immune system, followed by the endocrine system (27.3%, 3/11). The neurological systems showed the third highest level of gene expression (18.2%, 2/11), whereas the digestive and respiratory systems showed the lowest level (9.1%, 1/28) (Table 4). Our outcomes showed that the greater part of the overlapping genes was distributed in the hematologic/immune system, proposing that immunity may play a part in CIPN’s development. GOSemSim was used to distinguish the potential key genes. Based on the correlation index, the five highest results were selected as hub genes: *Cdh1*, *Fas*, *P2ry2*, *Satb2*, and *Zfhx2* (Figure 5A). To confirm and verify our sequencing and prediction data, we measured the expression levels of 5 hub mRNAs through RT-qPCR. The results showed that their expression in CIPN tissues was significantly changed compared with normal tissues, whereas the trend was consistent with the RNA-seq (Figure 5B).

### 3.5. Immune Infiltration Analysis

**Figure 6:**
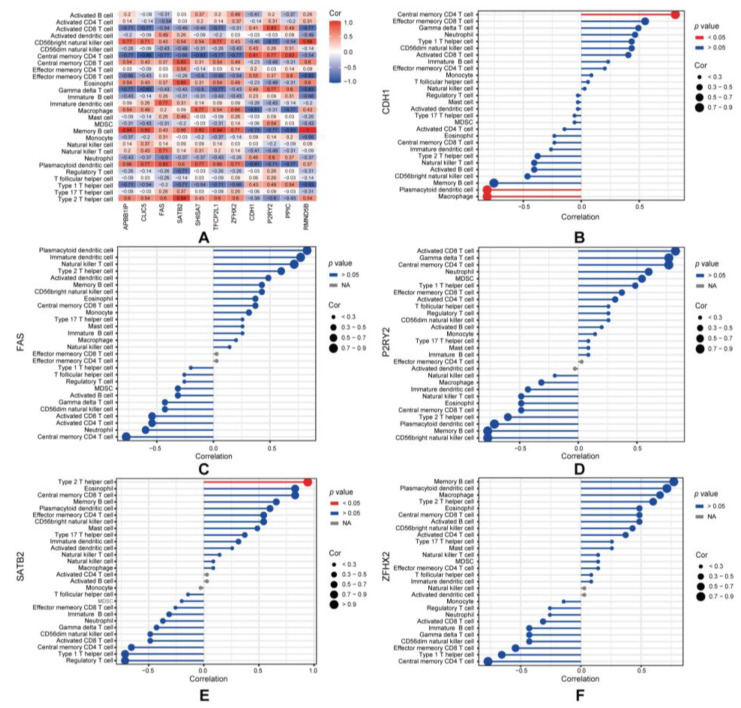
Analysis of correlation between overlapping mRNAs and immune cells. (**A**) The correlations between 11 overlapping mRNAs and immune cell infiltration in CIPN. (**B**–**F**) Correlations between CDH1 (**B**), FAS (**C**), P2RY2 (**D**), SATB2 (**E**), ZFHX2 (**F**) and infiltrating immune cells in CIPN.

We utilized CIBERSORT to evaluate the status of immune cell infiltration and subpopulations in CIPN rats and normal controls (Figure 5B,C). T cells CD4 memory, macrophages, and monocytes were found to be the main cells of interest. A correlation matrix was employed to display the proportion of different infiltrating immune cell subsets (Figure S2). Compared to normal controls, T cell CD4 memory resting infiltration was more prevalent in CIPN rats, while macrophages were less common. Moreover, the correlation analysis indicated a significant association between hub genes and plasma cells, monocytes, and T cells CD4 memory resting (Figure 5D), suggesting that they might play a key role in the immune regulations of CIPN. In order to diminish the error introduced by a single algorithm, we further evaluated the correlation between overlapping mRNAs and 28 immune cell types using ssGSEA and Spearman’s correlation analysis (Figure 6A). Surprisingly, we discovered that the hub genes were primarily correlated with macrophages, plasmacytoid dendritic cells, central memory CD4 T cells, and type 2 T helper cells in CIPN (Figure 6B–F). This result reaffirmed the importance of macrophages and CD4 T cells throughout the disease and indicated that dendritic cells, as well as type 2 T helper cells, were also potential mechanisms.

### 3.6. Prediction of Associated Diseases, Target Transcription Factors, and Drugs

**Figure 7:**
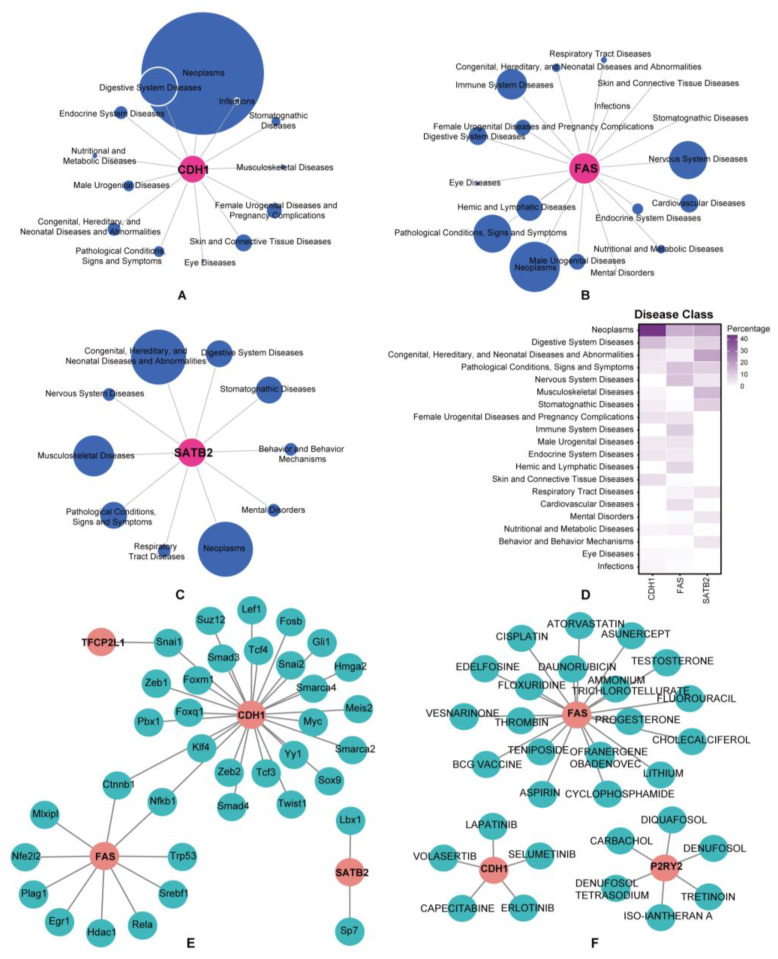
Disease-TF-drugs regulatory networks. (**A**–**C**) Disease Classes associated with hub genes. (**D**) Gene-Disease Class Heatmap. (**E**) TF prediction for hub genes based on the TRRUST database. (**F**) Drug-genes interaction network prediction. Red nodes represent hub genes. Blue nodes represent diseases, predicted TFs, or drugs.

We revealed the disease classes associated with the hub genes using the “disgenet2r” R package (Figure 7A–D). The results showed that *Cdh1*, *Fas*, and *Satb2* were strongly associated with neoplasms, whereas *Fas* was related to immune system diseases and nervous system diseases, hinting that they might have a stronger correlation with CIPN. According to the TRRUST database, 36 transcription factors (TFs) that may regulate hub genes were identified (Figure 7E), including *Ctnnb1*, *Fosb*, and *Foxm1*, laying the groundwork for further mechanistic research. Meanwhile, 30 potential drugs were anticipated to target the hub genes using the DGIdb database (Figure 7F), including Cholecalciferol, Erlotinib, and Aspirin. Cytoscape was utilized to visualize the TF genes and drug-gene interactions, thus providing new clues about the mechanisms and treatments for CIPN.

## 4. Discussion

Recently, CIPN has gained expanding attention owing to its associated health and economic burdens; however, the underlying molecular mechanisms remain unclear. According to previous studies, non-coding RNAs play significant roles in the development of CIPN. Li et al. identified that lncRNAs in the spinal cord, such as *LOC108353231*, *LXLOC_012126*, and *LXLOC_012123*, may mediate neuroinflammation and pain in CIPN [20]. Wang et al. showed that *miR-30d* participates in CIPN by downregulating *GAD67* [21]. Zhang et al. reported that *circ_0005075* deletion alleviated the progression of neuropathic pain by inducing *miR-151a-3p* and inactivating the *NOTCH2* signaling pathway [22]. Nevertheless, the interactions between non-coding RNAs and their associated regulatory networks require further investigation.

Previous studies have shown that inflammation and infiltration of immune cells are fundamental to Taxol-induced pain sensation and axonal damage [23]. Krukowski K found that paclitaxel-induced mechanical allodynia was more severe in T cell-deficient mice and that CD8+ T cells were necessary to resolve CIPN [24]. Zhang showed that paclitaxel stimulated innate immunity, bringing about the infiltration of macrophages into the spinal dorsal horns, which in turn prompted CIPN [25]. However, it remains unclear whether circRNAs influence immunity and mediate CIPN in a miRNA-dependent manner.

In this study, we used RNA-Seq and bioinformatics analyses to explore the gene expression profiles in the spinal dorsal horns of Taxol-treated rats. We screened 134 circRNAs, 353 lncRNAs, and 86 mRNAs that are differentially expressed in Taxol-induced CIPN. Next, we predicted the DE-circRNA-targeted miRNAs and then obtained 4004 mRNAs targeted by these miRNAs using TargetScan and miRDB. We examined the intersection of DE-mRNAs and predicted mRNAs and identified 11 overlapping mRNAs. Finally, we constructed circRNA-miRNA-mRNA regulatory networks containing 15 circRNAs, 18 miRNAs, and 11 mRNAs. Our results identified new circRNAs that might participate in CIPN development and explored their regulatory mechanisms as ceRNAs, providing fresh targets for treatment.

We further examined the miRNAs targeted by the DE-circRNAs. Among these miRNAs, *miR-125a-3p* negatively relates with the development and maintenance of inflammatory pain via regulating *MAPK* [26], *miR-324-3p* attenuates neuropathic pain through *circAnks1a*/*miR-324-3p*/*VEGFB* axis [27], and *miR-370-3p* promoted the inflammation and pyroptosis of spinal cord neurons by targeting *Caspase1* [28]. These results confirmed that the predicted circRNA-targeted miRNAs might play a crucial role in CIPN and that they likely functioned by regulating immunity.

To find out how the DE-mRNAs work, GO and KEGG enrichment analyses were conducted. Our results showed that both detected and predicted mRNAs were enriched in immune and inflammatory processes and pathways. Simultaneously, the overlapping mRNAs were enriched in the regulation of myeloid cell differentiation, response to cycloheximide, and cellular response to interleukin-1, which were associated with neuroimmune and neuropathic pain [29,30,31]. KEGG pathway analysis also illustrated that overlapping mRNAs were enriched in the p53 signaling pathway, natural killer (NK) cell-mediated cytotoxicity, and TNF signaling pathway, which could modulate the development of pain [32,33,34]. Moreover, NK cells, a key component of the innate immune response [35], regulate chronic pain and chemically induced neuropathies [36]. Together, immune and inflammatory responses might play a crucial part in CIPN, and immune-targeting therapies may be a potential option.

According to BioGPS, 11 overlapping genes are mainly enriched in the immune system, indicating the role of immunity in CIPN. The immune response in the nervous system supports not only the onset and progression of pain but also its resolution [37]. Activation of immune cells in damaged nerves leads to the release of pro- and anti-inflammatory cytokines as well as analgesics and analgesic mediators, which determine the process of pain [38]. Immune cells and their mediators, particularly nociceptive macrophages, support the maturation of neuroinflammation and contribute to paclitaxel-induced neuropathic pain [39]. Although there is growing recognition that neuroimmune communication is vital in CIPN pathogenesis, the specific mechanisms require further elaboration.

Next, we constructed a PPI network using GeneMANIA and applied GOSemSim to identify hub genes, including *Cdh1*, *Fas*, *P2ry2*, *Satb2*, and *Zfhx2*. Among the five hub genes, *Fas*, *Satb2*, and *Zfhx2* were upregulated, whereas *Cdh1* and *P2ry2* were downregulated in the spinal dorsal horns in CIPN.

*Cdh1* (Cadherin 1) encodes E-cadherin, a classical cadherin regulating cell–cell adhesions, tissue formation, and suppression of cancer [40]. Recent studies have revealed that adherens junctions are involved in neuroimmune interactions and chronic pain [41]. Li et al. found that upregulation of *Cdh1* attenuated neuropathic pain via inhibiting GABAergic neuronal apoptosis [42]. Consistent with our results, *Cdh1* expression was reported to correlate inversely with immune infiltration [43], including plasmacytoid dendritic cells and macrophages. However, further studies are required to identify the functions of cadherins in pain and neuroinflammation.

*Fas* (Fas cell surface death receptor) is a transmembrane receptor belonging to the tumor necrosis factor (TNF) receptor superfamily [44]. It plays a vital role in the physiological regulation of apoptosis and has been implicated in the pathogenesis of several diseases, especially in neoplasms and the immune system [45]. Functionally, Fas ligand-mediated apoptosis can promote neuronal apoptosis and chronic inflammation [46]. Moreover, the application of the Fas receptor suppresses post-traumatic neural degeneration and promotes functional recovery after spinal cord injury [47].

*P2ry2* (Purinergic receptor P2Y2) encodes nucleotide receptors mediating neurotransmission, the release of proinflammatory cytokines, and reactive astrogliosis [48]. Malin et al. indicated that nucleotide signaling through P2Y2 played a key role in thermal nociception [49]. Recent research has also shown that paclitaxel administration upregulated transient receptor potential vanilloid 1 (TRPV1) channels and purinergic receptors, resulting in hyperalgesia [50].

*Satb2* (SATB homeobox 2) encodes a DNA-binding protein that plays pivotal roles in development and tissue regeneration [51]. It regulates axonogenesis, synapse formation, and synaptic plasticity [52]. *Satb2* gene variants are associated with a multisystem neurodevelopmental disorder [53]. Nevertheless, *Satb2*’s function in pain pathogenesis and immunity is unknown.

*Zfhx2* (Zinc finger homeobox 2) is a transcriptional regulator highly expressed in differentiating neurons, critical for regulating pain perception and noxious stimuli [54]. *Zfhx2*-deficient mice showed increased depression-like behavior and anxiety-like phenotypes [55]. However, there is little evidence of its role in neuroinflammation. Our study further suggests an influential role in CIPN.

Neuroimmune crosstalk is of central importance in neuropathic pain, as it supports the initiation, progression, and resolution of pain [37]. Previous analyses have found that most immune cells, inflammatory mediators, cytokines, and chemokines were associated with neuropathic pain [38,56]. Activated immune cells such as macrophages, neutrophils, and lymphocytes can release cytokines and chemokines that typically induce pain [57]. Pain can also induce immunity and help sensitize nociceptors [58]. Consistently, we found that, in CIPN rats, there was a meaningful correlation between overlapping mRNAs and many immune cells. According to the immune infiltration analysis of hub genes, *Cdh1* mainly correlates with plasmacytoid dendritic cells and macrophages, which can modulate the function of nociceptor sensory neurons and the development of neuropathies [59,60]. *Fas* and *P2ry2* are negatively correlated with monocytes, *Satb2* is positively correlated with Type 2 T helper cells, and *Zfhx2* is closely related to T cells CD4 memory resting, suggesting their importance in CIPN and immunity.

TFs are DNA-binding proteins that identify and interact with specific DNA sequences to control genome expression, leading to various diseases [61]. Previous studies have revealed that TFs played a part in the pathophysiology of neuropathic pain and neuroinflammatory signaling [62]. We constructed TF-genes regulatory networks based on hub genes and identified 36 regulatory factors, including *Ctnnb1*, *Fosb*, and *Foxm1*, laying the groundwork for further exploration of the mechanism underlying CIPN.

Using the DGIdb database, 30 potential drugs were identified, including Cholecalciferol, Erlotinib, and Aspirin. Some of these drugs have been shown to have therapeutic potential for neuroimmune diseases. For instance, Cholecalciferol is mainly used to correct vitamin D deficiency, which is essential for metabolism, hormonal release, immune functions, and maintaining health [63]. The levels of calcium and calcitriol were reported to differ significantly between those who experienced chronic pain and those who did not [64]. Poisbeau et al. also found that Poisbeau helped to reduce rat neuropathic pain by modulating opioid signaling [65]. Erlotinib, an epidermal growth factor receptor (EGFR) inhibitor, has been used to treat several malignancies [66]. Kersten found that applying EGFR inhibition resulted in the relief of neuropathic pain via the *MAPK* signaling pathway [67]. Tretinoin (also known as retinoic acid) is a retinol (vitamin A) derivative used to treat photodamaged skin [68]. It could reduce neuropathy and promote neural regeneration [69], whereas retinoic acid signaling is essential for dendritic cell maturation and T cell immunity [70], consistent with our predictions. Although the efficacy of the putative drugs requires further validation, it may shed light on potential treatments and mechanisms for CIPN.

Nevertheless, several issues have been addressed in this study. First, some predictive results, such as miRNAs, TFs, and potential drugs, were obtained from various public databases and have limited significance. In addition, the expression of DE-circRNAs and DE-mRNAs warrants further validation and investigation. Finally, although bioinformatic analyses offer new insights into CIPN, the specific molecular mechanisms, and regulatory networks require further research.

## 5. Conclusions

In this study, 134 DE-circRNAs and 86 DE-mRNAs were screened, and circRNA-miRNA-mRNA regulatory networks associated with CIPN were constructed. We also identified *Cdh1*, *Fas*, *P2ry2*, *Satb2*, and *Zfhx2* as potential hub genes in CIPN. We further explored the immune microenvironment in CIPN, demonstrating the relationship between hub genes, DE-circRNAs, and the immune system, particularly the regulation of T cells and NK cell-mediated cytotoxicity, suggesting the importance of immunity in CIPN. The TFs regulatory networks and potential drugs were predicted to provide novel insights for further research and therapeutics.

## Figures and Tables

**Table 1:** Biological information for the top 20 upregulated circRNAs.

circRNA	Log2FC	*p*-Value	Regulation	Chrom	Strand	circRNA_Type	Gene Symbol
circRNA_08074	14.139014	0.002317	Up	Chr2	-	sense-overlapping	Map3k5
circRNA_10981	13.687971	0.000000	Up	Chr4	-	sense-overlapping	Zfp40
circRNA_17435	13.418825	0.000001	Up	ChrX	+	sense-overlapping	
circRNA_13195	13.364652	0.006881	Up	Chr6	-	sense-overlapping	Brsk2
circRNA_10018	12.959353	0.000062	Up	Chr3	+	sense-overlapping	Jak2
circRNA_12266	12.786437	0.044086	Up	Chr5	-	exonic	Pcgf5
circRNA_08650	12.434478	0.005765	Up	Chr2	+	intergenic	Pwwp2a
circRNA_08056	12.433880	0.001857	Up	Chr2	+	sense-overlapping	Rabep1
circRNA_03312	12.400673	0.002614	Up	Chr11	-	sense-overlapping	Acaca
circRNA_13652	12.391184	0.023951	Up	Chr6	+	sense-overlapping	Tex2
circRNA_17582	12.266018	0.004655	Up	ChrX	-	sense-overlapping	Arsg
circRNA_09053	12.260412	0.004717	Up	Chr2	-	exonic	Ttc3
circRNA_16414	12.243545	0.008860	Up	Chr9	-	sense-overlapping	Bbx
circRNA_01593	12.200506	0.023802	Up	Chr1	+	sense-overlapping	Opa1
circRNA_05160	12.188125	0.007071	Up	Chr15	-	sense-overlapping	Gtf2i
circRNA_08920	12.183688	0.007139	Up	Chr2	-	sense-overlapping	Zcchc2
circRNA_04866	12.068021	0.011526	Up	Chr14	+	sense-overlapping	Dpp10
circRNA_13511	12.020117	0.045608	Up	Chr6	+	sense-overlapping	Cep350
circRNA_07000	11.982601	0.025790	Up	Chr18	+	sense-overlapping	Lin54
circRNA_02033	11.981717	0.017243	Up	Chr10	+	sense-overlapping	Nop14

**Table 2:** Biological information for the top 20 downregulated circRNAs.

circRNA	log2FC	*p*-Value	Regulation	Chrom	Strand	circRNA_Type	Gene Symbol
circRNA_14081	−12.748110	0.046107	Down	Chr6	+	intergenic	
circRNA_04448	−12.422531	0.002173	Down	Chr14	+	sense-overlapping	Vom2r66
circRNA_11535	−12.340272	0.003422	Down	Chr4	+	sense-overlapping	Cntn6
circRNA_16338	−12.260065	0.030690	Down	Chr9	-	intergenic	
circRNA_16911	−12.245977	0.005434	Down	Chr9	-	exonic	Ncl
circRNA_03440	−12.150955	0.008460	Down	Chr12	+	sense-overlapping	Arhgef18
circRNA_12821	−12.150955	0.008460	Down	Chr5	+	sense-overlapping	Pum1
circRNA_14242	−12.148621	0.016776	Down	Chr7	+	intergenic	
circRNA_14478	−12.042250	0.013296	Down	Chr7	-	sense-overlapping	Trhde
circRNA_12787	−12.041095	0.013327	Down	Chr5	+	sense-overlapping	Csmd2
circRNA_11207	−12.040755	0.013336	Down	Chr4	-	sense-overlapping	Mkrn1
circRNA_16241	−12.040415	0.013345	Down	Chr8	+	sense-overlapping	Itga9
circRNA_02853	−12.039258	0.013376	Down	Chr10	+	sense-overlapping	Tnrc6c
circRNA_00395	−12.038917	0.013386	Down	Chr1	-	exonic	Qpctl
circRNA_00467	−12.035918	0.013467	Down	Chr1	-	sense-overlapping	Gpatch1
circRNA_07892	−11.928657	0.020688	Down	Chr2	+	sense-overlapping	Ssbp2
circRNA_05508	−11.921805	0.020954	Down	Chr15	+	sense-overlapping	Vwa8
circRNA_04328	−11.920180	0.021017	Down	Chr13	+	sense-overlapping	Cdc42bpa
circRNA_01155	−11.912544	0.036172	Down	Chr1	-	intergenic	
circRNA_14402	−11.912544	0.036172	Down	Chr7	+	sense-overlapping	Cep83

**Table 3:** Eleven hub DE-mRNAs between the results of prediction and sequencing.

Gene	Terms	log2FC	*p*-Value	Change
Apbb1ip	Amyloid beta precursor protein binding family B member 1 interacting protein	0.6285	1.87 × 10^−2^	Up
Clic5	Chloride intracellular channel 5	0.7067	2.25 × 10^−2^	Up
Fas	Fas cell surface death receptor	0.5995	3.08 × 10^−2^	Up
Satb2	SATB homeobox 2	0.8733	3.12 × 10^−2^	Up
Shisa7	Shisa family member 7	0.6166	1.17 × 10^−2^	Up
Tfcp2l1	Transcription factor CP2-like 1	0.5947	2.03 × 10^−2^	Up
Zfhx2	Zinc finger homeobox 2	0.6381	1.38 × 10^−2^	Up
Cdh1	Cadherin 1	−5.3748	1.45 × 10^−2^	Down
P2ry2	Purinergic receptor P2Y2	−0.7790	5.51 × 10^−2^	Down
Ppic	Peptidylprolyl isomerase C	−0.8716	1.98 × 10^−2^	Down
Rmnd5b	Required for meiotic nuclear division 5 homolog B	−3.1652	4.40 × 10^−2^	Down

**Table 4:** Tissue-most expressed genes identified by BioGPS.

System	Tissue/Cell	Genes
Hematologic/ Immune	Whole blood, Macrophage bone marrow, Mast cells	Apbb1ip, Fas, P2ry2, Rmnd5b
Neurologic	Nucleus accumbens, Dorsal root ganglia	Shisa7, Zfhx2
Digestive	Large intestine	Satb2
Respiratory	Lung	Clic5
Endocrine	Thyroid, Salivary gland	Cdh1, Ppic, Tfcp2l1,

## Data Availability

The data that support the findings of this study are available from the corresponding author upon reasonable request.

## Supplementary Materials

The following supporting information can be downloaded at: https://www.mdpi.com/article/10.3390/cimb45080430/s1, Figure S1: DE-lncRNAs identification; Figure S2: Correlation matrix among fractions of immune cell subtype; Table S1: Primer sequences used in RT-qPCR; Table S2: Prediction of circRNA-targeting miRNAs.
