# Supplementary material for: Identifying circRNA–miRNA–mRNA Regulatory Networks in Chemotherapy-Induced Peripheral Neuropathy
Source: Curr Issues Mol Biol. 2023 Aug 16;45(8):6804–22. doi: 10.3390/cimb45080430 (PMC10453290; doi:10.3390/cimb45080430)
Supplement: Supplementary file 1 [file cimb-45-00430-s001.zip › Supplematry.docx]

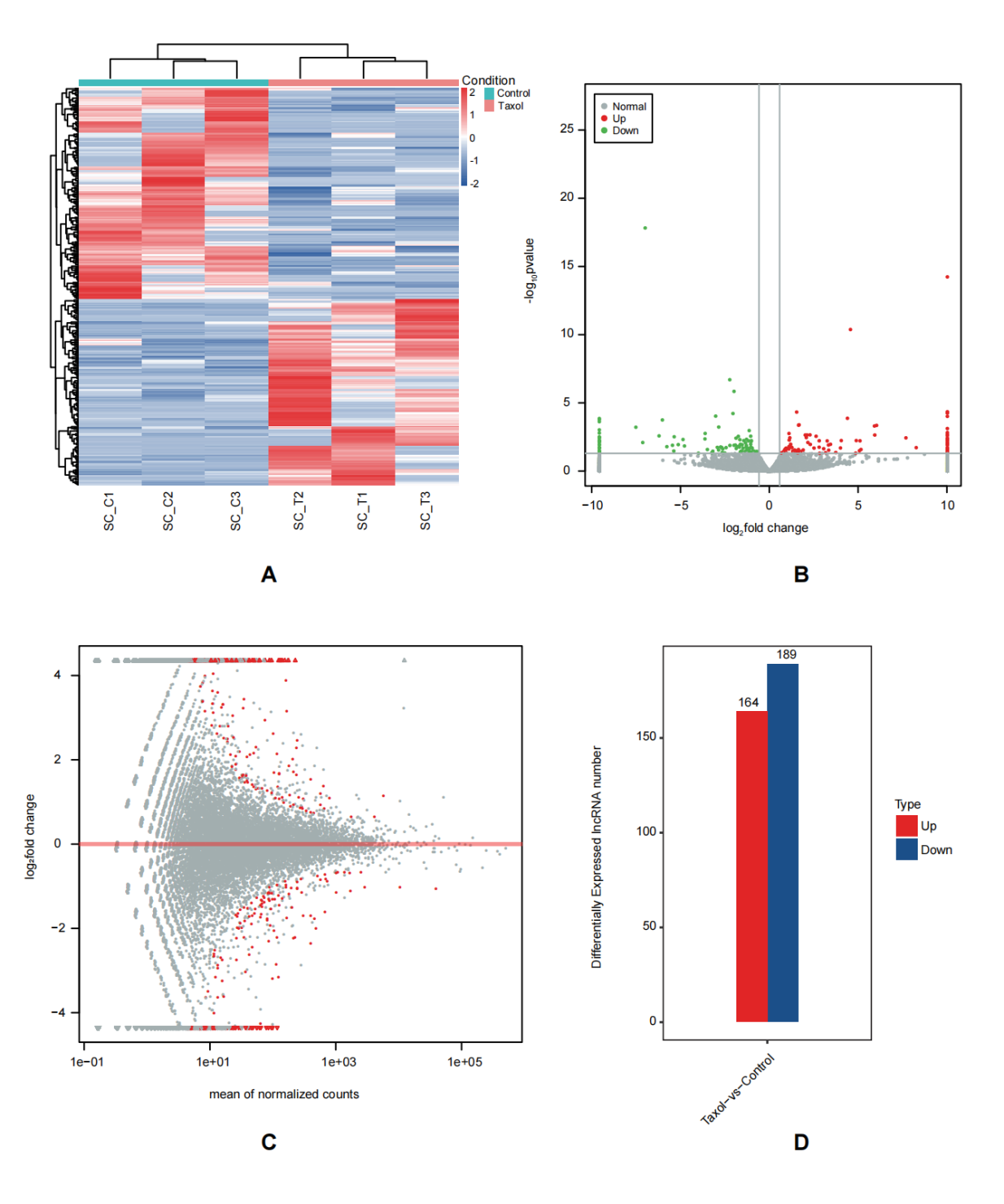


**Figure S1.** DE-lncRNAs identification. (A) The heatmap of the differentially expressed lncRNAs between CIPN and control samples. (B) Volcano plot of all DE- lncRNAs. Green dots indicate the downregulated lncRNAs, and red dots indicate the upregulated lncRNAs. (C) MA plot of DE-lncRNAs. (D) Statistics of DE-lncRNAs.


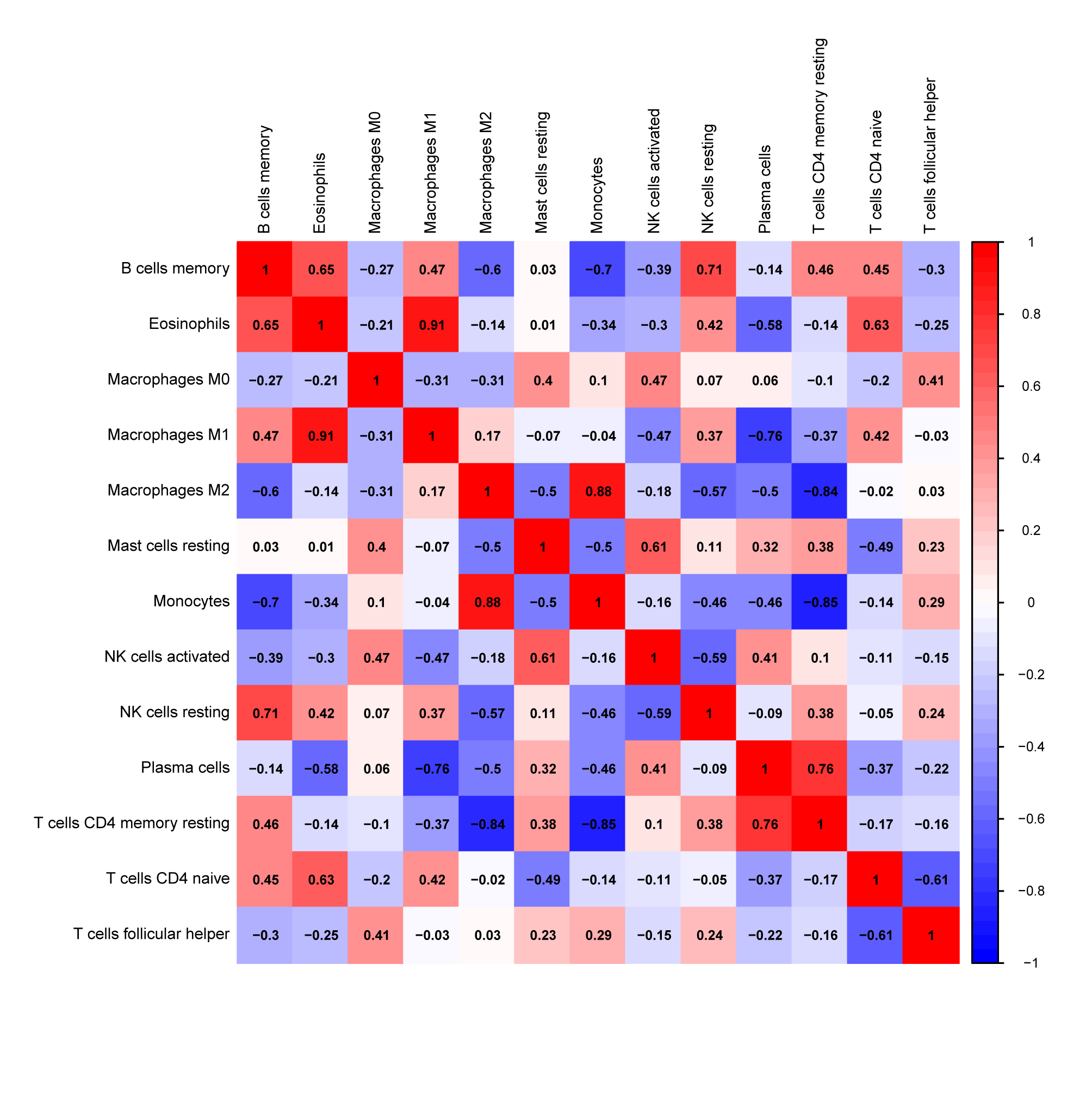


**Figure S2.** Correlation matrix among fractions of immune cell subtype.

**Table S1.** Primer sequences used in RT-qPCR.

| **Gene** | **Primer sequence (5′-3′)** |
| --- | --- |
| Cdh1 | \| Forward: CTGGGGTCATCAGTGTGGTC \| \| --- \| \| Reverse: TTGACCCTGGTACGTGCTTG \| |
| Fas | Forward: CCCGGACCCAGAATACCAAG  Reverse: TTCAAGTCCAC`AGGTGC |
| P2ry2 | Forward: CTGTCATGCTGGGTCTGCTT  Reverse: GCCAGTACTAAGGCGATGGT |
| Satb2 | Forward: TGATGATTCCGGTCTTCTGTGT  Reverse: CCAGCTTGATTATTCCTTGGGC |
| Zfhx2 | Forward: GGATGGGACTTCATACGGCG  Reverse: CCTTCCATTTCCAGCAGAAACTTAT |
| Gapdh | Forward: TGCCACTCAGAAGACTGTGG  Reverse: TTCAGCTCTGGGATGACCTT |

**Table S2.** Prediction of circRNA-targeting miRNAs.

| miRNA | circRNA |
| --- | --- |
| rno-miR-423-3p | circRNA_00068 |
| rno-miR-762 | circRNA_00333 |
| rno-miR-760-5p | circRNA_00467, circRNA_01155, circRNA_06379 |
| rno-miR-6322 | circRNA_00636 |
| rno-miR-330-5p | circRNA_00683 |
| rno-miR-1224 | circRNA_01155, rno-miR-6328 |
| rno-miR-17-5p | circRNA_01593 |
| rno-miR-324-3p | circRNA_01703, circRNA_06379, circRNA_15716 |
| rno-miR-760-3p | circRNA_01155, circRNA_02766, circRNA_12787, circRNA_14081 |
| rno-miR-298-5p | circRNA_02823, circRNA_02853, circRNA_06379, circRNA_13198 |
| rno-miR-3562 | circRNA_02853, circRNA_08650, circRNA_13706 |
| rno-miR-6334 | circRNA_03066 |
| rno-miR-6318 | circRNA_03440, circRNA_15716 |
| rno-miR-615 | circRNA_05275, circRNA_13198, circRNA_17088 |
| rno-miR-673-3p | circRNA_05641 |
| rno-miR-6328 | circRNA_06379 |
| rno-miR-3075 | circRNA_06475 |
| rno-miR-103-1-5p | circRNA_06880 |
| rno-miR-3065-3p | circRNA_08650, circRNA_14242 |
| rno-miR-147 | circRNA_11273 |
| rno-miR-412-3p | circRNA_11625 |
| rno-miR-1956-5p | circRNA_12532 |
| rno-miR-328a-5p | circRNA_06379, circRNA_12649 |
| rno-miR-3569 | circRNA_13511 |
| rno-miR-665 | circRNA_13545 |
| rno-miR-296-3p | circRNA_08650, circRNA_13706 |
| rno-miR-877 | circRNA_14242 |
| rno-miR-1247-5p | circRNA_14693, circRNA_16523 |
| rno-miR-125b-1-3p | circRNA_15371 |
| rno-miR-423-5p | circRNA_16414 |
| rno-miR-296-5p | circRNA_16523 |
| rno-miR-676 | circRNA_16979 |
| rno-miR-3541 | circRNA_01155 |
| rno-miR-345-3p | circRNA_01155 |
| rno-miR-351-5p | circRNA_02853, circRNA_17088 |
| rno-miR-370-3p | circRNA_02853 |
| rno-miR-339-3p | circRNA_02853 |
| rno-miR-486 | circRNA_02853 |
| rno-miR-6316 | circRNA_02853 |
| rno-miR-148b-5p | circRNA_02853 |
| rno-miR-3593-5p | circRNA_02853 |
| rno-miR-709 | circRNA_02853, circRNA_13706 |
| rno-miR-92b-5p | circRNA_06379, circRNA_15716 |
| rno-miR-331-3p | circRNA_06379 |
| rno-miR-188-3p | circRNA_06379 |
| rno-miR-673-5p | circRNA_06379 |
| rno-miR-339-5p | circRNA_06379, circRNA_12649 |
| rno-miR-503-5p | circRNA_06379 |
| rno-miR-667-3p | circRNA_06379 |
| rno-miR-3572 | circRNA_06379 |
| rno-miR-3573-5p | circRNA_06379 |
| rno-miR-667-5p | circRNA_06379, circRNA_16414 |
| rno-miR-6315 | circRNA_06379, circRNA_14242 |
| rno-miR-6333 | circRNA_06379 |
| rno-miR-532-3p | circRNA_06379 |
| rno-miR-21-3p | circRNA_06379 |
| rno-miR-207 | circRNA_06379, circRNA_08650 |
| rno-miR-320-3p | circRNA_08650 |
| rno-miR-125a-3p | circRNA_08650 |
| rno-miR-181b-5p | circRNA_08650 |
| rno-miR-466b-2-3p | circRNA_08650 |
| rno-miR-466b-4-3p | circRNA_08650 |
| rno-miR-466b-3p | circRNA_08650 |
| rno-miR-770-5p | circRNA_13198 |
| rno-miR-3072 | circRNA_13706 |
| rno-miR-540-3p | circRNA_13706 |
| rno-miR-874-3p | circRNA_14242 |
| rno-miR-23a-5p | circRNA_14242 |
| rno-miR-378a-5p | circRNA_14242 |
| rno-miR-702-3p | circRNA_14242 |
| rno-miR-500-3p | circRNA_14693 |
| rno-miR-935 | circRNA_15716 |
| rno-miR-3085 | circRNA_17088 |
| rno-miR-3084a-3p | circRNA_17088 |
